# Patient Safety Culture: Nurses’ Perspective in the Hospital Setting

**DOI:** 10.3390/healthcare12101010

**Published:** 2024-05-14

**Authors:** Maria José Reyes Ramos, Silvia Costa Abós

**Affiliations:** 1 Facultat d’Infermeria, Universitat de Barcelona, 08907 Barcelona, Spain; 2 Fundació Sanitària Mollet, 08100 Mollet del Vallès, Spain

**Keywords:** mixed method, nurses, patient safety, quality of healthcare, safety management

## Abstract

(1) Background: Patient safety culture (PSC) encompasses the values, attitudes, norms, beliefs, practices, perceptions, competencies, policies, and behaviours of professionals that determine organisational commitment to quality and patient safety. Few studies use mixed methods to analyse patient safety culture, and none offer the richness of using a mixed methodology to develop their theoretical model. This study aims to identify the factors nurses believe contextualise and influence PSC in relation to existing theoretical frameworks. (2) Methods: This study employed a sequential explanatory mixed-methods design combined with the Pillar Integration Process for data integration. (3) Results: In the final data integration process, 26 factors affecting nurses’ PSC were identified. Factors nurses related to PSC not being assessed with the tool used in phase 1 were notification system, flow of patients, patient involvement, resources and infrastructure, and service characteristics. (4) Conclusions: This mixed-methods study provides an opportunity to identify the weaknesses and strengths of currently developed theoretical frameworks related to PSC and offers content for its improvement. Even though multiple studies aim to assess PSC using existing quantitative method tools, the development of this study offers a glimpse of some aspects relevant to nurses’ PSC not included in the theoretical framework of the said tools, such as patient involvement, the flow of patients, and service infrastructure.

## 1. Introduction

Healthcare is intended to benefit individuals; yet in hospitals and highly specialised settings, it can cause harm. The complex combination of processes, technologies, and human interactions constituting modern healthcare delivery systems achieves significant benefits while, at the same time, entailing a risk of adverse events (AE) that occur all too often [1]. Safety could thus be defined as a state in which as few things as possible go wrong, referred to as Safety-I. In addition to ensuring the absence of incidents (or an acceptable level of risk), safety management must ensure that “as many things as possible go right”. This perspective is called Safety-II, and it refers to the ability of the system to function effectively under different conditions [2].

The concept of patient safety culture (PSC) encompasses the values, attitudes, norms, beliefs, practices, perceptions, competencies, policies, and behaviours of professionals that determine organisational commitment to quality and patient safety (PS) [3,4]. 

PSC is nowadays considered a priority in any healthcare system and is broadly studied through quantitative studies employing different measuring instruments for its evaluation. The most widely used tool for assessing safety culture is the Hospital Survey on Patient Safety Culture (HSOPSC) by the United States Agency for Healthcare Research and Quality [5]. This questionnaire assesses the perception of PSC among hospital staff. It is the most widely used tool in the European Union and is specific to hospitals [6]. The HSOPSC consisted of 42 questions grouped into a total of 12 dimensions, composed of 3 or 4 items per dimension. It was revised in 2019, and the second version currently consists of 32 questions grouped into 10 dimensions, providing reliability statistics based on quantitative data [5].

Through qualitative methodology, certain factors that can affect PS have been studied, such as lack of resources, use of new technologies, teamwork, or leadership [7,8,9,10]. There are few qualitative studies that analyse PSC globally [11]. 

One of the advantages of mixed-methods research (MMR), as explained by experts in mixed methods, is that the use of mixed methodology achieves a broader and deeper perspective of the phenomenon, providing an integral, complete, and holistic perception and obtaining a greater variety of perspectives on the object of study, namely, frequency, breadth and magnitude (quantitative), and depth and complexity (qualitative) [12,13,14,15]. In addition, MMR experts in PS have suggested that studies using a mixed-methods approach to assess safety culture would be helpful because they allow for the in-depth research needed to describe the multiple components of this construct [16]. There are very few studies analysing PSC with MMR, and all of them only compare the results obtained with a qualitative and quantitative methodology or deepen into the data obtained, ignoring the richness of the use of the mixed methodology to develop the theoretical model of PSC [17,18,19,20]. 

Thus, this study aims to analyse the PSC of nurses in a regional hospital in phase 1 by delving into the nurses’ perceptions of PS and the factors that, according to the nurses, contextualise and influence PSC in phase 2. The mixed approach allows the analysis of the PSC construct, with the final objective of this study being to identify the factors nurses believe contextualise and influence PSC in relation to existing theoretical frameworks.

## 2. Materials and Methods

### 2.1. Study Design

**Figure 1:**
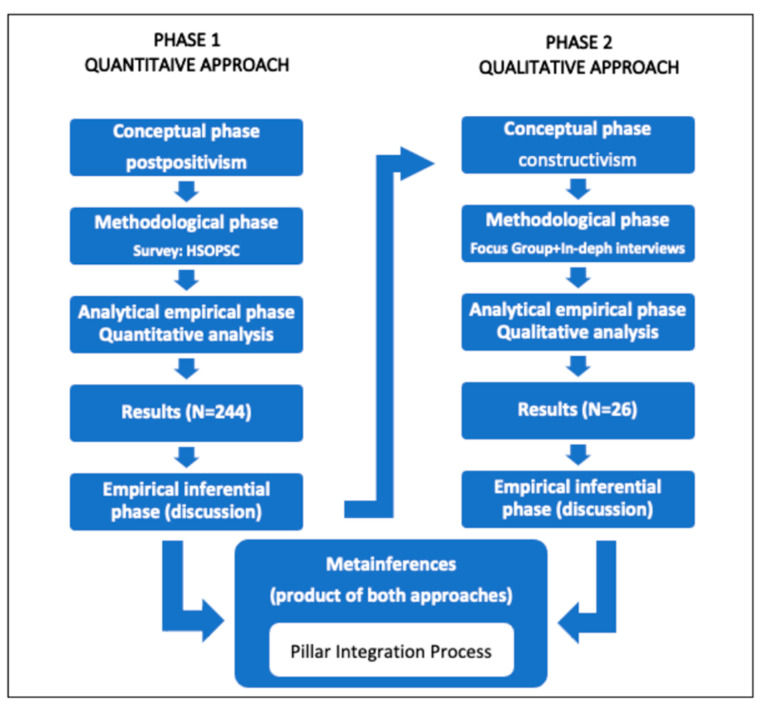
Mixed-methods sequential explanatory study design used in this study. Source: prepared by the authors based on Hernández Sampieri et al., 2010 [14].

The study’s design followed a sequential explanatory mixed-methods model. This design consists of a first phase in which quantitative data was collected and analysed to determine the nursing staff’s perception of the institutional PSC, followed by another phase in which qualitative data was collected and evaluated by delving into the nurses’ perceptions of PS and the factors that, according to the nurses, contextualise and influence PSC. Finally, the results obtained in both phases were integrated into the interpretation and elaboration of the study report to answer the final objective of the study, allowing for a more exhaustive discussion of the results [21,22]. In this final phase, the results of the two previous phases were integrated and interpreted to explain the factors contextualising and influencing nurses’ PSC by performing a Pillar Integration Process (PIP) [23]. Figure 1 summarises the study’s design, with the approach, sampling, and data collection and analysis methods used in each phase (Figure 1). 

This article details the relevant aspects of the mixed methodology. The design, methods, and results of each specific quantitative and qualitative study phase are described in other articles [24,25].

### 2.2. Study Population and Sampling

The study sample consisted of nurses working at the hospital in the different care areas at the time of data collection. In the quantitative phase, sampling was conducted by convenience and included all nurses working at the hospital at that time: 244 nurses (234 assistants and 10 nursing managers). The participation rate was 100%. The only inclusion criterion was that they had a current employment contract with the institution at the time of data collection [24].

For the recruitment of participants in the qualitative phase, a non-probability, intentional, and reasoned sampling system was used. The nurses were selected through a systematic procedure among those who completed the survey in the previous phase, a process that avoided the possibility of the selection of new participants causing inconsistencies in the inferences derived from the analysis of quantitative and qualitative data [26].

The systematic selection of participants in the second phase considered the findings of the previous phase as follows:

- Job position: The questionnaire used in the quantitative approach referred to those responsible for the management and direction of the hospital. Considering the significant differences in several aspects of the previous phase between manager and care nurses, a convenience sampling of all hospital manager nurses was performed.
- Unit/service of work: Informants of interest were selected for the purpose of the study. Among the care nurses, we selected those from the unit with the highest score in the dimension of the questionnaire that evaluates “openness in communication” and those from the units with the lowest and highest number of incidents reported in the last year. Nurses from the emergency department were also selected, as it was the service that rated the safety climate significantly lower than the rest.
- Working day, seniority, and work shift: Considering the statistically significant differences found in the previous phase, participants were selected from the different categories of each of these variables whenever possible.

The sample size was determined progressively throughout the research to achieve adequate representation in each focus group. Recruitment and focus groups continued until data saturation occurred. When saturation was is reached, no new information is generated in additional group data, which is a useful principle to determine sample size in a qualitative research study [27]. Aware of the limitations entailed when using focus groups to obtain data, two in-depth interviews were conducted that did not provide new data [28].

### 2.3. Data Collection

In the quantitative phase of the study, the HSOPSC questionnaire of the AHRQ, adapted and validated in the Spanish context, was used [29]. This questionnaire included 42 questions answered using a five-point Likert scale and grouped into 12 dimensions made up of three or four items per composite. Two dimensions referred to the hospital as a whole, and the rest focused on the unit or work area in which the respondent works. The questionnaire included the safety climate rating, represented through a subjective global appreciation of patient safety (from 0 to 10) and incident reporting during the last year (yes or no). Nine items used a five-point Likert scale to indicate the level of agreement (“strongly disagree” to “strongly agree”), and the other items used a five-point Likert scale to indicate frequency (“never” to “always”). Finally, the questionnaire asked for personal/professional factors, including sex, work shift, workday, job position, seniority, and unit or work area [24]. 

In phase 2, the data collection and analysis processes were designed to be emergent, circular, reflexive, and flexible. The data consisted of focus groups, in-depth interviews, and field notes taken during its implementation [25].

The strengths and opportunities for improvement, as well as the dimensions and the items with the lowest scores in the HSOPSC questionnaire found in the previous phase, were considered when adapting the research questions and preparing the second-phase data collection schedule. Because we worked with a series of focus groups as well as in-depth interviews, it was very important to maintain the structure and content of the questions, so all participants received the same questions and comparison was possible (see Appendix A).

### 2.4. Data Analysis and Integration 

Data analysis phase 1 used Jamovi 1.0.8.0 for MacOS. A descriptive analysis was made of all the variables included in the study. Categorical variables were summarised with their absolute and relative frequencies and continuous variables with their means and standard deviations (SDs). A bivariate analysis was conducted using contingency tables to study the relationship between different variables. Using the Kolmogorov–Smirnov test, it was found that the main variables (each of the dimensions of the questionnaire) did not follow a normal distribution. We studied the possible relationships between the main variables and personal and professional variables using the Mann–Whitney U test (for dichotomous variables) and the Kruskal–Wallis test (for categorical ones). The level of significance used in bilateral contrasts was *p* < 0.05 (alpha significance level of 5%) [24].

In phase 2, demographic and clinical information was summarised using descriptive statistics. After focus groups and interviews were transcribed, a content analysis was conducted. The study’s primary researcher (author 1) literally transcribed all the data collected. All transcripts were imported to ATLAS.ti 9 for data analysis, which combined inductive and deductive coding of categories and constant comparison between them [25]. Data collection and data analysis were made in the participants’ native language, and transcription and quotes were checked by participants to ensure the appropriate quality and validity criteria of the study [25]. Then, the first author, with the support of two experts on translation, translated quotes.

Data integration at the analytical and interpretation level was primarily conducted in two ways: (1) writing about the data in a discussion in which the separate results of the quantitative and qualitative analysis were discussed and (2) pre-submitting the data in a joint visualisation in table form, which simultaneously ordered the quantitative and qualitative results. This joint presentation is defined as a way to “integrate data by bringing it together through a visual medium to extract new insights beyond the information obtained from the separate quantitative and qualitative results” [30]. 

The approach to data analysis and integration in this study was interactive. Although quantitative and qualitative data were analysed sequentially, an interactive practice was adopted; both quantitative and qualitative study results were a source of information for each other to ensure consistency between the two lines of study and to achieve interpretive rigour and quality of the meta-inferences generated by the integration of both sets of results [26]. 

Thus, the findings of the qualitative phase led to an additional analysis of the quantitative results not considered a priori during phase 1, which provided a richer interpretation of both sets of data. Regarding the observation of the interaction between both study phases, the quantitative analysis was conducted first and considered only the highest score of the different items and dimensions (if it was positive or negative). Nevertheless, when analysing qualitative data, some concepts provided a different perspective compared to quantitative data. As a result, an additional analysis of each score obtained for each item and dimension was conducted to ensure the interpretation during data integration was correct. In this extra analysis, all scores (positive, negative, or neutral) were taken into account. For instance, while the dimension “Teamwork in the Unit/Service” was seen as positive in the first phase, in the qualitative phase it was considered an aspect to be improved and was categorised as “lack of teamwork”. This contradiction led to another analysis to differentiate the scores considered to be negative from those considered neutral in the questionnaire, both in the dimension as a whole and in each item comprising it.

Another purpose of using a sequential QUAN–QUAL mixed method was to improve the results with more complete data and more significant results, explaining unexpected or inconsistent findings obtained in the first phase of the study [31]. The core idea was that integrating quantitative and qualitative data would maximise the strengths and minimise the weaknesses of each type of data [32]. To ensure correct interpretations of the quantitative survey results—referring to the elaboration of unexpected quantitative results—it was decided that the reasons for the inconsistent scores found in the four dimensions presenting unacceptable internal consistency would be explored (<0.6). This led to an individual analysis of each item composing each of these dimensions, and the differences found in the next phase were explored. Therefore, the qualitative phase helped to understand the inconsistencies in these dimensions found in the quantitative phase.

**Figure 2:**
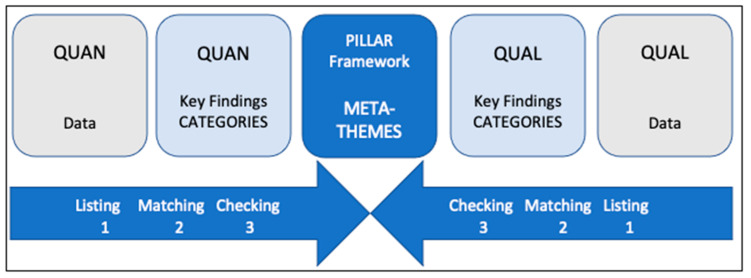
A generic diagrammatic representation of the Pillar Integration Process to demonstrate column headings and the direction of integration. Source: prepared by the authors based on R. E. Johnson et al. (2017) [23].

The integration of the results sought to identify factors contextualising and impacting nurses’ PSC in some way. To this end, an integrated analysis guided by a Pillar Integration Process (PIP) was conducted (Figure 2) [23]. This process consists of four stages to integrate qualitative and quantitative findings and present them in a joint display: (1) listing, (2) matching, (3) checking, and (4) pillar building. The pillar represents the meta-themes, similar to meta-inferences, and it appears as a central column in their joint display.

Stages 1–3 of the four-stage PIP were conducted deductively, so the HSOPSC dimensions provided the pillars of analysis. In the PIP first stage, raw quantitative and qualitative data and key findings were listed. In the second stage, the contents of the quantitative and qualitative lists were matched with the HSOPSC pillar dimensions. In the third stage, they were cross-checked for completeness and appropriate matching. In the fourth and final stage, pillar building, the joint display was analysed to create meta-inferences that provided a more exhaustive understanding, explanation, and context of the factors influencing hospital nurses’ PSC. This analysis made it possible to generate a visual illustration of the said factors, proving that the process by which the factors were related to PS can influence the hospital nurses’ PSC.

### 2.5. Scientific Rigour

In this study, scientific rigour was ensured independently for quantitative and qualitative approaches, seeking validity and reliability for the former and appropriate quality and validity criteria for the latter. Checklists, including recommendations for study reporting, were used to improve the quality of the reporting in each of the phases and the assessment of the strengths and weaknesses of the said phases. Thus, the STROBE statement checklist for cross-sectional observational studies was used for the quantitative approach. For the qualitative approach, the COREQ checklist was used for explicit and complete reports of qualitative studies using in-depth interviews and focus groups [33,34]. 

Specific indicators have been considered to evaluate the validity and quality of the mixed design, among which interpretive rigour, design quality, and legitimacy are worth mentioning. In addition to ensuring that this study incorporates all the aspects mentioned by Harrison et al. (2020) [35], strategies defined by Ivankova (2014) [36] for a sequential explanatory QUAN–QUAL mixed-methods design were applied in order to guarantee that the integrated conclusions of sequentially generated quantitative and qualitative findings are plausible. Ivankova [36] defines a three-step procedure: applying a systematic process to select participants for qualitative follow-up, adding detail to unexpected quantitative results, and observing the interaction between qualitative and quantitative lines of study. These have been previously detailed in the Sampling and Data Analysis and Integration sections, respectively.

### 2.6. Ethical Considerations

The study complied with the principles of the Declaration of Helsinki and the guidelines of the Ethics Committees for Human Research, ensuring good clinical practice and applicable legislation, with prior approval by the Ethics Committee for Scientific Research of reference in our centre.

All participants received the relevant information about the study in writing through an informative document with the correct explanation for their complete understanding before their inclusion in the project. All participants were informed that participation was voluntary and that they could withdraw at any time for any reason and with no consequences. The consent of the participants was obtained in writing prior to their inclusion in the study. By means of this informed consent, the participants gave their approval to be filmed and recorded and approved the use of the information obtained for research purposes. During the entire process of the study and in the future, the anonymity of all participants was and is guaranteed, as is the confidentiality and security of the information. Identification codes were used in the data collection and in interview transcription to ensure the confidentiality of the participants.

To guarantee freedom of speech on phase 2 and avoid any possible coercion in the participants’ narration, author 2, who conducted all focus groups and interviews, was external to the institution and is trained in qualitative research methods. To encourage participants to speak freely, she listened carefully and respectfully during the interview. Nursing managers participated as an exclusive group to prevent their presence from interfering with the other participating nurses’ opinions, provided that issues related to management and leadership were addressed. 

## 3. Results and Integrated Findings

The study participants were nurses who worked at the hospital when data collection occurred. For the quantitative phase, 244 nurses were recruited from all hospital units (response rate = 100% of the total number of nurses working at that time in the hospital). For the qualitative phase, a subsample of 26 consenting survey participants participated in four focus groups and two in-depth interviews. A non-probabilistic, intentional, and reasoned sampling system was used to guarantee the representation of the personal and professional variables of the participants in the quantitative phase of the study.

First, before data integration, the description of the sample of participants in both phases was taken into account to guarantee rigorous and valid data integration (Table 1):

- The nurses participating in the qualitative phase belonged to the three care areas with the highest representation in the survey and the care area with the lowest representation. That way, it was possible to obtain representativeness of the variability of the discourses.
- As for the independent variables described in the quantitative phase, each appeared in a similar proportion among the participants in phase 2. Regarding work positions, the nursing managers’ opinions were incorporated.

The PSC-related results from both study phases were then integrated. The data were structured following the 12 dimensions of the questionnaire used in the first phase of the study and the two additional questions regarding the degree of safety and incident reporting. The results of both phases can be found in Appendix B, which contains the results of the quantitative phase (in Table A2), for each item and for each dimension as a whole. Some of the verbatim responses related to the results of the qualitative phase can be found in Table A3. Moreover, because the quantitative phase was based on the Spanish version of the HSOPSC questionnaire, the following criteria were applied to analyse the results obtained in the first phase of the study [24]. Note that the positive response rate for each domain was calculated, with scores of 4 or 5 on the Likert scales being considered positive; negatively worded items were reverse coded before calculation. If the percentage of positive responses for one domain was ≥75%, this was considered an area of strength for patient safety culture. If the percentage of negative responses (scores of 1 or 2 on the Likert scales) for one dimension was ≥50%, this was considered an area of weakness [24].

### 3.1. Frequency of Events Reported

This dimension included three items referring to the perception of the frequency of reporting three types of events or mistakes not causing adverse effects (those detected and rectified before reporting, those likely to have caused damage but did not, and those predictably not harmful).

The results of the respondents are described in Table A2. It can be seen that most of the answers were positive. The figure is quite far from the 75% needed to consider it a strength, both for the dimension and for each item.

In phase 2, the nurses confirmed they only report incidents they consider to be serious and to have consequences for patients. Furthermore, they distinguished between notifying the incident on the institutional reporting system and communicating it to team members, but this always occurred on the basis that they were informing only about those incidents that had affected the patient in any way (see verbatim No. 1 in Table A3).

Nonetheless, this dimension cannot be deemed an opportunity for improvement according to the negative results obtained in phase 1, which are far from the 50% needed to consider a dimension as such. This result might be related to the perception, shared by all groups in the second phase, of the advantages of notifying an AE, such as avoiding incident repetition, seeking solutions, and even helping the professional who made the mistake (see verbatim No. 2 in Table A3).

### 3.2. Overall Perception of Safety

This dimension included four items, all aimed at measuring whether there is a perception of working in a way that jeopardises PS. Two of them referred to specific causes, such as the pace of work and the effectiveness of existing procedures to avoid mistakes in clinical care.

According to the results obtained during the quantitative phase (see Table A2), overall, this dimension was not an aspect needing improvement. Nevertheless, there was one item that ought to be improved: the one related to the idea that work pace can eventually affect patient safety. In this item, the negative responses exceeded 50% (specifically, 59.4%), which is the limit established to identify the item as a weakness or an opportunity for improvement in the safety climate.

The results obtained in the qualitative phase highlighted the pressures to which nurses are exposed while working. They believe they are working under pressure and coping with a heavy workload, which puts PS at risk. Nurses from the emergency department added that the nurse/patient ratio increases the risk of making mistakes. Moreover, when referring to workload, nurses included performing tasks that were not part of their job (see verbatim Nos. 3 and 4 in Table A3).

Regarding existing procedures, there was a silent majority giving a positive score to the item. In the qualitative phase, interviewees specified that having standardised procedures benefits PS (see verbatim No. 5 in Table A3).

Finally, the item regarding the avoidance of mistakes just by luck was graded positively by 43.5% of respondents. Nurses expressed it in the qualitative phase with the words “a guardian angel” who prevents more AE from happening (see verbatim No. 6 in Table A3). 

### 3.3. Supervisor/Manager Expectations and Actions Promoting Patient Safety

This dimension encompassed four items representing the perception respondents have of the importance service managers and supervisors give to PS. Neither the dimension nor any of its items could be considered a strength of the safety culture, as the percentage of positive answers was not ≥75%. Nonetheless, more than half of the respondents saw the dimension positively. During phase 2, nurses identified the support provided by their supervisor, and they described it as feeling heard. Moreover, they felt like they were receiving help, both to fix their mistake and to take measures to prevent it from happening again (see verbatim No. 7 in Table A3). 

Furthermore, nurses stated that when the workload increased during the pandemic, their supervisor assumed care activities that were not his/her responsibility. This situation would explain that the item “Whenever pressure builds up, my supervisor/manager wants us to work faster, even if it means taking shortcuts (negatively worded)” had a high number of negative answers (see verbatim No. 8 in Table A3).

### 3.4. Organisational Learning/Continuous Improvement

This dimension included three items about the proactive attitude towards PS. Respondents positively rated having access to activities to improve PS and adopting suitable measures to avoid error repetition. Nevertheless, the item referring to the evaluation of the changes already made to better PS was more diverse, with the same percentage of neutral and positive answers (see Table A2).

In all the groups in phase 2, except that of the emergency department, interviewees considered it positive that there is training in aspects of PS. They stated that although training is provided, there should be more, and it should be offered in every area of PS. The most veteran nurses believed PS was now part of the care process compared to previous years. This data corroborated that most respondents think there are activities aimed at improving PS (see verbatim No. 9 and 10 in Table A3).

When sharing their experience during the qualitative phase, nurses stated that making mistakes is a source of learning. They think reporting errors is crucial to introducing measures preventing them from happening again (see verbatim No. 11 in Table A3).

### 3.5. Teamwork within Units/Services

Though it cannot be considered a strength, this dimension received the highest score in the quantitative phase, especially for the items referring to respect and support shown by the staff (see Table A2). Nonetheless, in the qualitative phase, negative aspects regarding teamwork stood out. For instance, the risk of making mistakes increases when several professionals attend to one patient or when someone helps an overworked professional. Nurses mentioned the lack of effective communication among team members at the expense of PS. In the Emergency Department, they highlighted that teamwork was a positive aspect for PS during the pandemic (see verbatim No. 12, 13, and 14 in Table A3).

### 3.6. Communication Openness

In this dimension, the questionnaire results revealed a slight majority of respondents can speak freely about something that may affect PS and are not scared of asking questions when something has been done incorrectly (see Table A2). In the qualitative phase, nurses confirmed this aspect and assessed positively being able to talk to other team members and nursing managers to improve PS (see verbatim No. 15 in Table A3).

### 3.7. Feedback and Communication about Error

This dimension comprised three items about the information professionals receive when an error occurs or is reported and whether their unit discusses how to prevent it from happening again. Of all three, the lowest-rated item was the one regarding the lack of information on what type of action is taken when an incident is reported (see Table A2). Participants in phase 2 mentioned this lack of notification of the measures taken when there is an incident, their continuity, and the errors taking place in their service/unit (see verbatim No. 16 in Table A3).

### 3.8. Nonpunitive Response to Errors

This dimension did not reach the 75% response frequency necessary to be considered a strength entirely. Nevertheless, the item stating “Staff feel like their mistakes are held against them” was one of the most favourably rated in the questionnaire, with 70.5% of positive responses (see Table A2). In the qualitative phase, nurses referred to non-punitive responses as the main characteristic of PSC, and they defined the concept of “second victim” and the need not to blame the professional. In contrast, they showed self-punitive feelings when facing AE, such as shame, fear, and remorse (see verbatim No. 17 and 18 in Table A3). 

### 3.9. Staffing

The results of the quantitative phase showed this dimension as an aspect to be improved, with 62.5% of negative responses (see Table A2). One of the items comprising this dimension, specifically “We work in ‘crisis mode’ trying to do too much, too quickly”, was the worst rated in the whole questionnaire. In the qualitative phase, nurses detailed this situation, and their statements suggested that staffing affects the global perception of safety. They believed the institution should improve two more items: “We have enough staff to handle the workload” and “Staff in this unit work longer hours than is best for patient care” (see verbatim No. 19 and 20 in Table A3).

Nonetheless, during the quantitative phase, the item “We use more agency/temporary staff than is best for patient care” did not reach the 50% of negative responses needed to consider it an aspect to be improved in this dimension. After the first wave of the pandemic, nurses argued that the fact that the nursing team was, one year later, made up of more junior nurses with less experience than usual was a risk for PS. The COVID-19 era, hence, is a clear example of how the lack of personnel negatively influences PS (see verbatim No. 21 in Table A3).

### 3.10. Hospital Management Support for Patient Safety

This dimension included three items, two of which were considered by respondents opposingly in terms of management involvement. Whilst “Hospital management provides a work climate that promotes patient safety” obtained 42.2% of negative responses, “The actions of hospital management show that patient safety is a top priority” obtained 43% of positive answers (see Table A2). In addition, it should be noted that the former got a very low number of positive responses in the questionnaire (28.3%), only behind two items of the previous dimension (2 and 5, with 19.3% and 16.4%, respectively).

In the qualitative phase, nursing managers appealed to the engagement of hospital management regarding assistance to second victims and to the need to introduce a care programme for professionals affected by an AE. They did not go into detail about the management support for PS, although interviewees highlighted some aspects that indirectly allude to the involvement of hospital management and that are tackled in the following chapter (see verbatim No. 22 in Table A3).

### 3.11. Teamwork across Units

The four items comprising this dimension did not reach 50% of positive responses, and the lack of coordination among hospital units stood out negatively with 41.4% of responses. Nevertheless, most respondents believed it was not uncomfortable to work with staff from other services/units and that the different services cooperate and coordinate with each other. 

In general, these data contradicted the information gathered during the qualitative phase. Said information showed that nurses believe working with personnel from other services/units during the first wave of the pandemic had a negative impact on PS. Considering the Surgical Area was the worst rated during the quantitative phase, this information agrees with the fact that nurses belonging to this area were the ones transferred to other services during the pandemic to a greater extent (see verbatim No. 23 in Table A3).

### 3.12. Handoffs and Transitions

In this dimension, the negative score regarding the transition of patient information between different services slightly stood out, although it was not considered to be problematic (see Table A2). In the qualitative results, nurses believed the transition of information during shift changes is a risk for PS when referencing the pandemic and that it is a risk when the professional is inexperienced (see verbatim No. 24 in Table A3). 

### 3.13. Degree of PS in a Service/Unit

The mean score for the PS degree obtained during phase 1 was 6.69 ± 1.71 out of 10, with the emergency department scoring significantly lower (5.57 ± 1.67) (see Table 2). The differences between services regarding nurses’ perceptions of the factors related to PS during the qualitative phase highlight the lower degree of PS perceived in this department compared to the rest of the hospital units.

Below are all the elements that, during the qualitative phase of this study, were considered by nurses to have a different impact in the emergency department compared to the other services:

- Nurse/patient ratio: Compared to other services, the nurse/patient ratio in the emergency department is unstable. Hence, the number of patients per nurse increases, and as a result, this unit copes with higher levels of excessive healthcare workload.
- Interruptions and distractions: Nurses in the emergency department linked them to the presence of the patients’ families, considering their absence as an aspect favouring PS during the pandemic.
- Infrastructure: Despite limited space, there are no restrictions regarding the number of patients accessing the service. This element was mentioned when referring to the pandemic.
- Work organisation: With the ideal for nurses being that each nurse takes care of one patient and performs one task at a time, all groups considered the emergency department as the least organised. Even during the pandemic, they deemed it positive that they could work in a more organised way due to isolation measures.
- Therapeutic relationship: Like in the surgical area, nurses considered that the emergency department does not provide individualised or patient-centred care that favours patient engagement for their safety.

The nurses of the emergency group were the only ones who did not identify some PS-favouring aspects mentioned in other groups, such as training in subjects related to PS or the availability of standardised protocols and processes/procedures. Nonetheless, it was the only group in which professional experience was considered a factor favouring PS.

### 3.14. Written Notification of Any Incident Related to Patient Safety in the Past Year

A total of 82% of nurses answered this question, saying they had not reported any incidents during the past year, with significant differences regarding the unit/service, their work position, seniority, and working day (see Table 3 and Table 4).

During the qualitative phase, nurses referred to the need to use an incident reporting system to improve PS, emphasising that all professionals are involved in PS. It is worth mentioning some elements that may influence the nurses’ lack of AE reporting:

- The unawareness by some nurses of the institution’s incident reporting system, despite deeming it accessible to all professionals.
- The complex and time-consuming form, which nurses do not have enough time to complete.
- The notification of incidents being considered by nurses to be of greater risk, either because they entail negative consequences for the patient or because they have immediate repercussions that need to be solved. Hence, when their mistakes do not affect the patient in the end, when they do not have consequences, or when the said consequences are not severe, nurses do not report them. Although already mentioned, it is a crucial factor to be considered.
- The fact that the mistake to be notified is made by another team member, which stops nurses from reporting AE.
- The nurses’ lack of knowledge of whether what happened was an AE related to PS or not and, as a result, whether they should report it. In line with this lack of knowledge of what is considered an incident to be reported, it is worth mentioning the diversity of incidents identified by nurses and the different types in each group. Nursing managers are the nurses who report the most—80% according to the quantitative phase results (see Table 3)—and are the group to have determined the most AE during the qualitative phase. They stated in the focus group that the non-identification of certain events as safety problems, such as bedsores, causes them not to be reported.
- The uncertainty of whether the reporting system is anonymous makes care nurses cautious when it comes to reporting some AE (a factor shared by nursing managers).

Table 3 shows that nurses in the inpatient unit reported some incidents significantly more than the rest of the services (33.3%), and, regarding seniority, nurses with more than five years of seniority reported considerably more incidents than the most junior nurses (21.9% vs. 43%). In the qualitative phase, results showed that, in the inpatient unit, junior professionals are considered to have more difficulties admitting their mistakes than experienced nurses.

Moreover, senior nurses expressed that there has been an increase in the importance of PS and its recently acquired relevance, fostering a culture of prevention of avoidable patient harm and making professionals aware of the risks and the possibility of making mistakes during the care process. 

Full-time nurses reported more frequently than part-time nurses (24.3% vs. 7.6%) (Table 3). In the qualitative phase, there was no specific data regarding the working hours. This low reporting frequency among part-time nurses might be related to the nurses’ belief that the more time spent at the patient’s bedside, the greater the possibility of making a mistake.

Lastly, the authors integrated and merged the data using deductive PIP. The integrated results revealed which aspects and how they influence nurses’ PSC in relation to the theoretical framework used for its assessment through quantitative methods (specifically the HSOPSC). 

The integration of the data obtained 26 factors influencing nurses’ PSC, identified as pillars in this process: service/unit, professional experience, work position, work shift, identifying preventable problems, notification system, workload, protocols and action guides, chance, leadership, staffing, training, communication, teamwork, information, non-punitive response, exhaustion, professional experience, management involvement, patient flow, interruptions/distractions, infrastructure, patient involvement, technological and material resources, confidence to report AE, and AE types.

Appendix C shows the integration of the data obtained and the factors influencing nurses’ PSC, identified as pillars in this process, some of which are not specifically addressed in the HSOPSC (Table A4).

## 4. Discussion

Following data integration, the factors contextualising nurses’ PSC are discussed in the context of current literature, showing the processes by which PS-related factors can influence nurses’ PSC.

Reporting on PS-related incidents is linked to a positive PSC since it shows the professionals’ commitment to identifying preventable problems and their confidence to report them to improve PS. Nurses report incidents they consider to be serious and to have consequences for patients. Individuals possessing high levels of psychological safety are crucial to effective and safe healthcare delivery and the promotion of organisational learning. Such individuals contribute by discussing risk and adapting to avoid error; consequently, the organisation can find new pathways and processes to facilitate future positive outcomes [37]. Nurses do not report incidents that do not affect the patient or whose consequences are not severe. Speaking up about safety concerns could prevent future harm to other patients, improve safety systems, and contribute to a learning organisation. Some elements identified in this study could be considered motivations to speak up, such as avoiding incident repetition, seeking solutions, and even helping the professional who made the mistake. More research is needed to identify voicing factors and barriers, find strategies to avoid this silence, and promote psychological safety [38].

Incident reporting is related to Safety-I, as it aims to determine what went wrong (causes and factors leading to the incident) and learn from it. Given the results of this study regarding incident notification, it should be considered how PSC takes into account Safety-II, which aims to understand and adopt work as it is done to obtain optimal results adapted to each situation. [2,39].

When assessing incident reporting as an attribute of PSC, factors related to the notification system itself should be considered, such as its accessibility or the ease vs. complexity of using the system. All this is in addition to contemplating other more handy means of notification, such as verbal notification of situations reported by professionals to their colleagues or superiors, even if no written communication is made [40]. Nursing managers are the nurses who report the most. This goes along with Schwappach and Richard’s study, which confirms associations between clinical function and, thus, hierarchy, with being more likely to speak up and less likely to withhold their voice compared with other professional groups [38].

Furthermore, besides considering a non-punitive PSC that fosters blame-free attitudes and considers errors as systemic, it is essential to contemplate the self-punitive feelings experienced by nurses, such as shame or guilt [41,42]. 

PSC should consider whether nurses receive information on AEs that have occurred in their unit and the measures adopted to prevent the detected risks [43,44,45,46,47]. Likewise, another factor to be taken into account is the availability of information related to what is considered a reportable situation and to good practices. Training in good practices and topics related to PS is essential, including guidance and information on the notification system and its purpose [48,49,50,51]. 

One of the most relevant factors for PS is workload concerning the nurse/patient ratio, the overload due to a lack of staff, the increase in the pace of work due to this overload, or the tasks to be performed by the nurse in addition to her own. Regarding staffing, the staff’s experience in a particular area or service should be considered [10,52]. 

Teamwork should be considered a significant element for all it entails, especially in relation to non-technical skills, such as communication between professionals, trust, and leadership [53]. In addition to considering teamwork in the service and between hospital units, interprofessional work (between nurses) must be differentiated from intraprofessional work (with the rest of the professionals in the team) in terms of communication and organisation in the team [10,54,55,56,57]. 

Leadership is another crucial element in that team leaders must promote and prioritise PS throughout the process, both in avoiding risks, providing support when there is a safety problem, and implementing the necessary measures to improve PS. Moreover, this leadership must facilitate communication so that professionals can speak freely about PS and feel heard [10,58,59]. 

Coordination between units is an element that, besides what has already been mentioned about team communication, encompasses the flow of patients between services or within the same service. High patient flow is another factor considered to put PS at risk, although in our study, unlike in others, this problem is not attributed to the centre’s management [60].

Regarding patient flow, it is necessary to put each service in context. For instance, accident and emergency departments are units that differ from the other hospital areas in different aspects: the amount of human and technical resources is always the same despite workload fluctuation, professionals endure high levels of stress, the pace of work is demanding, and there is a high influx of patients [61].

Likewise, the patient’s own characteristics are another factor to consider since several care complexity individual factors are associated with certain AE [62]. 

On patient involvement, multiple studies address patient participation in PS, as well as the therapeutic relationship with respect to the humanisation of care or person-centred care, as an essential factor for PS [63,64,65,66,67]. Nonetheless, existing questionnaires to measure patient participation in PS still have some limitations in the active engagement of patients and family members [68]. 

Regarding resources and infrastructure and their impact on PS, several studies refer to the technological and material resources influencing PS [7,69,70]. Others even include water quality in the concept of infrastructure (in terms of infection prevention [71]) as well as functional aspects such as lighting, heating, and air conditioning, and even the condition of the tiles, which can pose a tripping or falling hazard if they are broken or loose [72]. There are no studies identifying what should be the optimal structural characteristics for safe care, a factor considered by nurses as incident to PS.

### Limitations

A limitation of this study on PSC is that it explores PSC only in nurses, which may not adequately capture the complex multidisciplinary nature of PSC in a healthcare setting. Future mixed-methods studies should be conducted to obtain evidence from other professionals and form a complete representation of PSC in hospitals. 

Another limitation related to participants is that the research is based solely on the perceptions and experiences of the nurses working in a specific hospital. Hence, the information provided responds to the nurses’ perceptions in the context in which the study was conducted and cannot necessarily be extrapolated to other settings. We aimed to delve into the reality perceived by nurses in terms of the factors affecting PSC.

The third limitation to be considered is the tool used in the quantitative phase of the study for data collection because the application of another questionnaire might have yielded different results. Other tools aim to measure perceptions of PS in terms of work satisfaction, teamwork, working conditions, or perception of management (such as the Safety Attitudes Questionnaire [73]), but the HSOPSC was chosen because it is the most widely used in the European Union and is specific for hospitals.

## 5. Conclusions

This study has made it possible to assess and understand hospital nurses’ perceptions of PSC by integrating and merging quantitative and qualitative data. 

The results obtained in the first phase of this study did not differ from the data obtained in quantitative studies carried out in other centres; however, they highlighted the need to conduct an in-depth investigation of the meanings that nurses give to their experiences with PSC and the need to know what they attribute this perception of PSC to.

The quantitative results were consistent with the qualitative results. While the quantitative analysis revealed significant shortcomings in the different dimensions of PSC, the qualitative results provided detailed information on the factors influencing these shortcomings and allowed us to delve deeper into aspects related to PSC. Thus, this mixed-method study has allowed us to explore the processes by which PS-related factors may influence nurses’ PSC. 

Even though multiple studies seek to assess PSC using existing quantitative method tools, the development of this study has provided a glimpse of some aspects relevant to nurses’ PSC not included in the theoretical framework of these tools, such as patient involvement, the flow of patients, or service infrastructure. Future research should study the inclusion of these elements in the PSC assessment tools as well as their impact on PS.

## Figures and Tables

**Table 1:** Relative frequencies of personal/professional variables in both phases of the study.

Variables	Categories	Results Phase 1 (*n* = 244)	Results Phase 2 (*n* = 26)
Sex	Woman	214 (87.7%)	23 (88.5%)
	Man	30 (12%)	3 (11.5%)
Unit/Work Area	Outpatient services	13 (5.33%)	
	Surgical area	51 (20.90%)	7 (26.9%)
	Inpatient units	67 (27.46%)	2 (7.7%)
	Mother and child area	20 (8.20%)	
	Emergency department	68 (27.87%)	5 (19.2%)
	Support services	14 (5.74%)	
	Mental health and addictions	11 (4.51%)	5 (19.2%)
Working day	Full-time	152 (62.3%)	18 (69.2%)
	Part-time	92 (37.7%)	8 (30.8%)
Work Position	Care nurse	234 (95.90%)	19 (73%)
	Nursing manager	10 (4.10%)	7 (26.9%)
Seniority	<2 years	23 (9.42%)	2 (7.7%)
	2–5 years	34 (13.93%)	5 (19.2%)
	>5 years	187 (76.65%)	19 (73%)
Work Shift	Morning	126 (51.6%)	14 (53.8%)
	Afternoon	69 (28.3%)	7 (26.9%)
	Night	49 (20.1%)	5 (19.2%)

Source: prepared by the authors.

**Table 2:** Results for the question “Rate from 0 to 10 the degree of safety in your service/unit” presented by care areas.

“Rate from 0 to 10 the Degree of Safety in Your Service/Unit”	Mean	Standard Deviation
Outpatient services	6.77	2.01
Surgical area	6.92	1.35
Inpatient unit	7.03	1.53
Mother and child area	7.75	1.41
Emergency department	5.57 *	1.67
Support services	7.5	1.70
Mental health and addictions	7.45	1.57

* *p* < 0.001. Source: prepared by the authors.

**Table 3:** Results for the question “During the past year, you have reported in writing any incident related to patient safety” presented by care areas.

“During the Past Year, You Have Reported in Writing Any Incident Related to PS”	% Yes	% No
Outpatient services	23.1	76.9
Surgical area	5.9	94.1
Inpatient unit	31.3 *	68.7
Mother and child area	10	1.41
Emergency department	16.2	83.8
Support services	21.4	78.6
Mental health and addictions	9.1	90.9
TOTAL	18	82

* *p* < 0.001 according to Kruskal–Wallis test. Source: prepared by the authors.

**Table 4:** Affirmative results for the question “During the past year, you have reported in writing any incident related to patient safety” presented by seniority, working day, and job position.

“During the Past Year, You Have Reported in Writing Any Incident Related to PS”			
SENIORITY	<2 years	2–5 years	>5 years
%YES	4.3	5.9	21.9 *
WORKING DAY	Full-time	Part-time	
%YES	24.3 *	7.6	
JOB POSITION	Nursing Manager	Care Nurse	
%YES	80 *	15.4	

* *p* < 0.05. Source: prepared by the authors.

## Data Availability

All data are available from the corresponding author on reasonable request. Throughout the study process and in the future, the anonymity of all participants is guaranteed, as is the confidentiality and security of the information. The system recorded which nurses answered the questionnaire in the first phase, but the content of the responses was anonymous, assigning a random number to each participant. In the second phase of the study, identification code numbers were used in the data collection and in the transcription of the interviews to ensure the confidentiality of the participants.

## Appendix A

The following table shows the interview and focus group guide question statements to address in the second phase regarding the first-phase results (Table A1).

**Table A1:** Questions to address in the second phase regarding the first-phase results.

1st Phase Results	HSOPSC Dimension—Item	Theme	Question
Strengths	Dimension 5. Teamwork in the Unit/Service	Experience before an AE	What has been your experience with an AE?
	Item 4. In this unit, we all treat each other with respect	Experience before an AE	What has been your experience with an AE?
	Item 1. Staff support each other	Support or help received	What support or help have you received?
	Item 8. If colleagues or superiors find out you’ve made a mistake, they use it against you	Factors Influencing AE Reporting	Why are some AE notified and others not?
Opportunities for improvement	Dimension 9. Staffing	Experience before an AE	What factors influence PSC?
	Item 5. Sometimes the best patient care cannot be provided because the working day is exhausting	Experience before an AE	What factors influence PSC?
	Item 14: We work under pressure to get too done too quickly	Experience before an AE	What factors influence PSC?
	Item 2. There are enough staff to cope with the workload	Experience before an AE	What factors influence PSC?
	Item 15: Never increase the pace of work if it means sacrificing PS	Experience before an AE	What factors influence PSC?
Discrepancies between units/services	Item “rate from zero to ten the degree of PS in your service/unit”	Factors influencing PS	What factors influence PSC?
	Item “during the last year has reported in writing an incident related to PS”	Factors Influencing AE Reporting	Why are some AE notified and others not?

HSOPSC: Hospital Survey on Patient Safety Culture; PS: patient safety; PSC: patient safety culture; AE: adverse event. Source: prepared by the authors.

## Appendix B

Main results of both phases. The results of the quantitative phase can be found in Table A2, both for each item and for each dimension as a whole. Some of the verbatim responses related to the results of the qualitative phase can be found in Table A3.

**Table A2:** Results of PSC on the HSOPSC questionnaire by items and dimensions.

	% of Negative Answers	% of Neutral Answers	% of Positive Answers	Mean Score (Standard Deviation)
40. When a mistake is made but is detected and corrected before affecting the patient, how often is this reported?	17.6	31.2	51.2	3.45 (1.01)
41. When a mistake is made but has no potential to harm the patient, how often is this reported?	20.5	34.8	44.7	3.31 (1.01)
42. When a mistake is made that could harm the patient but does not, how often is this reported?	20.5	30.7	48.8	3.37 (1.03)
Total dimension “Frequency of Events Reported”	19.53	32.20	48.23	3.37 (0.94)
10. It is just by chance that more serious mistakes don’t happen around here	31.6	25	43.4	2.81 * (1.09)
15. Patient safety is never sacrificed to get more work done	59.4	16	24.6	2.51 (1.12)
17. We have patient safety problems in this unit	38.5	23.8	37.7	2.96 * (1.16)
18. Our procedures and systems are good at preventing errors from happening	21.7	25.8	52.5	3.32 (0.99)
Total dimension “Overall Perceptions of Patient Safety”	40.55	22.45	37	2.90 (0.78)
19. My supervisor/manager says a good word when he/she sees a job done according to established patient safety procedures	12.3	27.5	60.2	3.53 (0.87)
20. My supervisor/manager seriously considers staff suggestions for improving patient safety	17.2	21.3	61.5	3.52 (0.98)
21. Whenever pressure builds up, my supervisor/manager wants us to work faster, even if it means taking shortcuts (negatively worded)	58.2	26.6	15.2	3.59 * (1.0)
22. My supervisor/manager overlooks patient safety problems that happen over and over	11.4	19.7	68.9	3.78 * (0.96)
Total dimension “Supervisor/Manager Expectations and Actions Promoting Patient Safety”	14.05	23.78	62.20	3.60 (0.76)
6. We are actively doing things to improve patient safety	13.9	17.6	68.5	3.62 (0.91)
9. Mistakes have led to positive changes here	16	17.6	66.4	3.59 (1.0)
13. After we make changes to improve patient safety, we evaluate their effectiveness	26.2	36.9	36.9	3.08 (0.91)
Total dimension “Organizational Learning-Continuous Improvement”	18.70	24.03	57.23	3.43 (0.74)
1. People support one another in this unit	11.5	13.9	74.6	3.80 (0.90)
3. When a lot of work needs to be done quickly, we work together as a team to get the work done	17.6	18.1	64.3	3.62 (1.02)
4. In this unit, people treat each other with respect	11.1	13.1	75.8	3.89 (0.91)
11. When one area in this unit gets really busy, others help out	15.6	21.7	62.7	3.57 (0.90)
Total dimension “Teamwork Within Units”	13.95	16.88	69.15	3.72 (0.76)
35. Staff will freely speak up if they see something that may negatively affect patient care	7	27	66	3.77 (0.87)
37. Staff feel free to question the decisions or actions of those with more authority	25.4	38.9	35.7	3.10 (0.96)
39. Staff are afraid to ask questions when something does not seem right	54.5	32	13.5	3.50 * (0.94)
Total dimension “Communication Openness”	15.30	32.63	15.30	3.46 (0.70)
34. We are given feedback about changes put into place based on event reports	23.4	38.9	37.7	3.17 (1.03)
36. We are informed about errors that happen in this unit	15.2	34	50.8	3.47 (0.96)
38. In this unit, we discuss ways to prevent errors from happening again	10.2	34	55.8	3.55 (0.89)
Total dimension “Feedback and Communication About Error”	16.40	32.50	48.07	3.39 (0.73)
8. Staff feel like their mistakes are held against them	70.5	22.9	6.6	3.88 * (0.86)
12. When an event is reported, it feels like the person is being written up, not the problem	47.2	22.1	30.7	3.20 * (1.10)
16. Staff worry that mistakes they make are kept in their personnel file	32.8	24.2	43	2.88 * (1.05)
Total dimension “Nonpunitive Response to Errors”	26.60	23.10	50.27	3.32 (0.76)
2. We have enough staff to handle the workload	68	12.7	19.3	2.27 (1.08)
5. Staff in this unit work longer hours than is best for patient care	16.4	10.6	73	2.21 * (1.04)
7. We use more agency/temporary staff than is best for patient care	43.9	20.5	35.6	3.10 * (1.10)
14. We work in “crisis mode” trying to do too much, too quickly	13.5	13.5	73	2.13 * (1.03)
Total dimension “Staffing”	62.40	14.35	23.28	2.43 (0.76)
23. Hospital management provides a work climate that promotes patient safety	42.2	29.5	28.3	2.76 (1.03)
30. The actions of hospital management show that patient safety is a top priority	29.1	27.9	43	3.12 (1.06)
31. Hospital management seems interested in patient safety only after an adverse event happens	35.2	27.5	37.3	2.95 * (1.06)
Total dimension “Hospital Management Support for Patient Safety”	36.20	28.43	35.37	2.95 (0.87)
24. Hospital units do not coordinate well with each other	31.1	27.5	41.4	2.84 * (1.01)
26. There is good cooperation among hospital units that need to work together	28.7	22.5	48.8	3.20 (0.99)
28. It is often unpleasant to work with staff from other hospital units	63.1	24.2	12.7	3.67 * (0.91)
32. Hospital units work well together to provide the best care for patients	17.3	27	55.7	3.41 (0.88)
Total dimension “Teamwork Across Units”	25.10	25.10	49.78	3.28 (0.66)
25. Things “fall between the cracks” when transferring patients from one unit to another	44.3	18.9	36.8	3.09 * (1.01)
27. Important patient care information is often lost during shift changes	43.4	24.6	32	3.15 * (0.99)
29. Problems often occur in the exchange of information across hospital units	52.5	25.8	21.7	3.36 * (0.96)
33. Shift changes are problematic for patients in this hospital	47.1	27.9	25	3.27 * (0.93)
Total dimension “Handoffs and Transitions”	28.80	24.50	46.73	3.22 (0.73)

* After changing the format of the questions formulated in negative. Source: Prepared by the authors.

**Table A3:** Results on phase 2: Verbatims.

Verbatim Number	Related Verbatim
1	“On paper, I think we’re registering very little…and I think there are mistakes we ignore thinking ‘we don’t need to record it because it’s not serious enough”, and we just register those we think ‘whops, that was quite a slip-up’ or ‘it has caused a fall resulting in death or whatever’. In those cases, we do report it on paper because we believe it’s too serious, and we need to try to avoid it”.
2	“Patient safety culture means you inform, but you do it to get some improvement, not to punish someone. I think that’s something we’ve improved over time”.
3	“With the amount of work we have right now and the lack of staff, the situation is overwhelming. It’s just impossible to do everything, and the problem of not being able to do everything is what she was saying, that the bigger your ratio is, the more problems you can have on, you know, adverse events of many things”.
4	“And you solve stuff that maybe is not strictly your job, it doesn’t concern you (…) But to get where you want to be, you must do that job because otherwise, you won’t get there. For example, it’s like you’re your own secretary, picking up the phone all day when you should be concentrating on what you need to concentrate on. Or now send me this paper, now phone the lab, now there’s a label missing, now send that for me, now this has been left somewhere, now take this person to this medical test, now (puff) that person shat himself…”
5	“I think protocols have helped a lot to avoid mistakes. Checklists…”
6	“But they are factors that, I don’t know, we have a guardian angel… don’t we?”
7	“Yes, managers are ready both to prevent things from happening and, if they do happen, to be there for you so you know it’s a team. She’s your manager, and she’s going to be on your side, and she’s not going to tell you: ‘You messed up, you’re sacked’; no, she tells you ‘I’m with you, let’s see what happened, how we can fix it, what can we do so it doesn’t happen again’. She does a good job”.
8	“I think the workload quadrupled. I remember one night counting 74 people being admitted to the ED and only five or six nurses there. Even the manager came and took care of 10 patients”.
9	“There *is* training. The thing is it should have more importance”.
10	“Now patient safety is more of a priority than before”.
11	“The moment we admit we make mistakes, it might help us see other types of solutions or to not repeat our mistake again”.
12	“Because the doctor doesn’t work at the same pace as the nurse (…) You team up a lot, but there are too many hands involved, and that’s the thing, if someone has loaded something that didn’t have to be loaded, that is injected in 100 cc and instead they’ve administered it as a bolus, I don’t know that, because I haven’t seen it”.
13	“You see it in the example I was talking about, about the stretcher-bearer who took the woman… who was not the woman. Nobody told the nurse he was taking the woman… when she goes to the OR, no one…. probably, if communication was more fluid… maybe not, maybe it could not have been avoided, but… It’s a factor that maybe could be improved, the communication within the team”.
14	“We have a lot of teamwork. I know if I need help, I’ll have it even without asking for it. I know, for example, in the 12-h shifts, it was always the same people (…) I know how they work. I know that if my co-worker loads a Nolotil for me, she’s not going to put it in a bolus. It’s the trust that comes from many years working together (…) That’s what I think. I see it as positive for patient safety”
15	“Once, I administered the wrong medication, and I must admit my manager, my colleagues, and the doctor treated me very well: they gave me support, and we fixed it, and that was all. You can feel pretty calm. You don’t have someone telling you off and saying: ‘What have you done? You administered that, and this is not how you do it!’. On the contrary, you only hear: ‘Okay, this happened, let’s see what solutions we can find’. You monitor your patient, the doctor is with you, and, honestly, I felt very good. Despite what may or may not have happened, I had support”.
16	“If there has been any adverse event, (…) then they should allow staff to do that, to communicate it. And if something has happened to me or one of my colleagues, then we need to have the tools to fix it, to be aware of it. In some way, people will have absorbed this idea and will be aware of what is happening at all times, or they will bear it in mind (…) For some time, our supervisor has been giving us the meetings in writing, which provides us with a lot of information. Before, this didn’t happen, but our current supervisor now gives us the information on paper. This is an advantage we could use in our favour”.
17	“You don’t blame anyone, because we all have learned you have to listen, to try to come up with solutions, and then try to prevent it from happening again…”
18	“(…) and the shame of making a bad impression, of others telling me ‘wow, you’re such a bad nurse’ because I don’t know how to do that when it’s something very easy to do”.
19	“With the amount of work we have right now, and the lack of staff, it’s just too much. It’s impossible to do everything. And the problem of not being able to do everything is what she was saying, that the higher your patient ratio is, the more problems you can have on, you know, adverse events of all kinds of stuff”.
20	“I’m going to tell you something, it’s not I’m trying to make excuses, but the pressure we have right now a lot of times leads to… I don’t even know how we don’t make more mistakes. We have so much pressure I don’t know how we don’t make more mistakes. It’s often impossible to cope with the number of operations in one morning. And this often makes you work hurriedly. For some years now, the caseload has been huge. And, well, I think there could be even more errors than there are. We have very few”.
21	“There are people who have started, who were finishing their studies, and they put them to work suddenly (…) And that overloads the rest of their co-workers. Everyone has to learn, obviously, but… not only the issue of handling things, but the issue of managing stress (…) It’s not the same”
22	“It would be a great support to have psychological help or a second victim unit, neither of which we have in this centre. (…) I sometimes find myself alone because I don’t have tools to solve the AE that might occur during my service”.
23	“They took us out of our usual job and put us in other places we didn’t know at all, where we didn’t have a team we knew (…) For me the feeling has been I’ve been to war”
24	“(…) It’s not the same for someone new as for someone who has been working for a long time and knows how to control it, place it, although they cannot cope with everything (because you simply can’t). It’s impossible to have each patient where they have to be, to know what is in each place. It’s not the same… You only learn how to handle everything with time and through experience. It’s true it’s not the same when you’re getting reports of all patients and at the same time you don’t know where patient files are, or what kind of patient you’re treating…”

Source: Prepared by the authors.

## Appendix C

The following table shows the integration of the data obtained and twenty-six factors influencing nurses’ PSC, identified as pillars in this process, some of which are not specifically addressed in the HSOPSC (Table A4).

**Table A4:** Pillar Integration Process results.

Quantitative Data	Key Results in Phase 1 (Quantitative)	Pillars	Key Results in Phase 2 (Qualitative)	Qualitative Data
Service/work unit	Significant differences in bivariate analysis: service/unit and 8 dimensions	Service/Unit	In the emergency focus group (FG) they did not identify some favourable aspects for PS that arose in other FG	Category: factors favouring PS Subcategory: have protocols
			The emergency department is the least organised	Category: factors favouring PS Subcategory: organization of work
			In the emergency department and surgical area, an individualised or person-centred care that favours the participation of the patient in their safety is not provided	Category: factors favouring PS Subcategory: therapeutic relationship
Seniority	Significant differences in bivariate analysis: seniority and 5 dimensions	Professional experience	Professional experience favours PS, although attention may decrease, increasing the risk of errors	Category: factors considered ambivalently Subcategory: professional experience
Work position	Significant differences in bivariate analysis: work position and 5 dimensions	Work position: Healthcare or Management	Nurse managers’ report more types of AE	Category: types of AE
Work shift	Significant differences in bivariate analysis: work shift and 3 dimensions	Work shift		
Mistakes that have no potential to harm the patient are reported (44.7% positive responses)	1. Reporting patient safety events % positive responses: 48.23 % neutral responses: 32.20 % negative responses: 19.53	Identifying preventable problems	Incidents considered to be serious and have consequences for patients are reported.	Category: AE teatures Subcategory: severity or consequences of AE
Mistakes that are caught and corrected before affecting the patient are reported (51.2% positive responses)		Notification system	Incidents are reported verbally to the rest of the team	Category: AE features Subcategory: severity or consequences of AE
The pace of work is never rushed if it involves sacrificing PS (59.4% negative responses)	2. global perception of security % positive responses: 37 % neutral responses: 22.45 % negative responses: 40.55	Workload	Work is done in a hurry and under pressure, and there is a high workload	Category: factors not favouring PS Subcategory: care overload
Our procedures and systems are good at preventing errors (52.5% positive responses)		Protocols and action guides	The existence of protocols favours PS and prevents variability in clinical practice	Category: factors favouring PS Subcategory: have protocols
No more mistakes occur by chance (43.4% positive responses)		Chance	Having an “angel” that prevents more AE	Category: factors not favouring PS Subcategory: inadequate structural resources
My supervisor/manager overlooks PS problems that happen over and over (68.9% negative responses)	3. Supervisor/manager expectations and actions promoting PS % positive responses: 62.20 % neutral responses: 23.78 % negative responses: 14.05	Leadership	Receive support from the unit supervisor: feel heard and receive help, both to solve a mistake and to put measures in place to prevent it from happening again	Category: support received Subcategory: managers
My supervisor/manager wants us to work faster during busy times, even if it means taking shortcuts (58.2% negative responses)		Staffing	Given the work increase during the pandemic, the supervisor assumed care activity	Category: factors affecting PS (during the pandemic) Subcategory: lack of staff
We are actively doing things to improve patient safety (68.5% positive responses)	4. Organisational learning/continuous improvement % positive responses: 57.23 % neutral responses: 24.03 % negative responses: 18.70	Training	Training in PS aspects as a positive but insufficient aspect	Category: factors not favouring PS Subcategory: lack of training
Mistakes have led to positive changes here (66.4% positive responses)		Training	Making a mistake is a source of learning (communicating it to adopt measures to prevent it from happening again)	Category: implications for nurses Subcategory: find solutions
Staff support each other (74.6% positive responses)	5. Teamwork in the unit/service % positive responses: 69.15 % neutral responses: 16.88 % negative responses: 13.95	Communication	Lack of effective communication within the team to the detriment of the PS	Category: factors not favouring PS Subcategory: lack of teamwork
When a lot of work needs to be done quickly, we work together as a team to get the work done (64.3% positive responses)		Teamwork	When several professionals attend to the same patient, the risk of mistake increases	
During busy times, staff in this unit help each other (62.7% positive responses)		Workload	When someone helps the professional who is overloaded, the risk of mistake increases	
		Teamwork	Stable teamwork with mutual trust as a positive aspect for the PS	Category: impact on PS during the pandemic
Staff speak up if they see something that may negatively affect patient care (66% positive responses)	6. Communication openness % positive responses: 52.07 % neutral responses: 32.63 % negative responses: 15.30	Confidence to report AE	Being able to talk to teammates and nurse managers to improve PS is positively valued	Category: support received Subcategory: from colleagues
Staff are afraid to ask questions when something does not seem right (54.5% negative responses)				Category: support received Subcategory: from managers
We are informed about changes that are made based on event reports (37.7% positive responses)	7. Feedback and communication about error % positive responses: 48.07 % neutral responses: 32.50 % negative responses: 16.40	Information	Lack of information about what kind of actions are carried out when an incident occurs and their continuity, as well as the errors that occur in the service/unit	Category: factors not favouring PS Subcategory: lack of information
Staff feel like their mistakes are held against them (70.5% negative responses)	8. Non-punitive response to errors % positive responses: 50.27 % neutral responses: 23.10 % negative responses: 26.60	Non-punitive response	Non-punitive response to errors as a main characteristic of PSC. Nurses define the concept of second victim and the need not to blame the professional. In contrast, they narrate self-punitive feelings about AE, such as guilt, shame, fear, and remorse	Category: implications for nurses Subcategory: feelings
We work in “crisis mode” trying to do too much, too quickly (73% positive responses)	9. Staffing % positive responses: 23.28 % neutral responses: 14.35 % negative responses: 62.40	Workload	The most mentioned element in terms of negative impact on PS is patient load	Category: factors not favouring PS Subcategory: work overload
We have enough staff to handle the workload (68% negative responses)		Staffing		
Staff in this unit work longer hours than is best for patient care (73% positive responses)		Exhaustion		
This unit relies too much on temporary, float, or PRN staff (43.9% negative responses)		Professional experience	After the first wave of the pandemic, the fact that the nursing team consists of a higher number of novice nurses with less experience than usual is a risk for PS	Category: factors affecting PS (during the pandemic) Subcategory: lack of staff
Hospital management provides adequate resources to improve PS (42.2% negative responses)	10. Hospital management support for patient safety % positive responses: 35.37 % neutral responses: 28.43 % negative responses: 36.20	Management involvement	Currently, PS is present in the care process	Category: PS concept Subcategory: evolution of PS
The actions of hospital management show that PS is a top priority (43% positive responses)			Management should implement support programs for the 2nd victims	Category: support received Subcategory: from management
Hospital units do not coordinate well with each other (41.4% positive responses)	11. Teamwork across units % positive responses: 49.78 % neutral responses: 25.10 % negative responses: 25.10	Patient flow	Working under pressure to relieve care pressure from other services	Category: PS during the pandemic
It is often unpleasant to work with staff from other hospital units (63.1% negative responses)		Service/Unit Experience	Working with staff from other services/units during the first wave of the pandemic had a negative impact on PS	Category: factors affecting PS (during the pandemic) Subcategory: lack of staff
Shift changes are problematic for patients in this hospital (47.1% negative responses)	12. Handoffs and information exchange % positive responses: 46.73 % neutral responses: 24.50 % negative responses: 28.80	Communication	The transfer of information between shifts during the pandemic and when professionals were inexperienced is considered a risk to PS	Category: factors affecting PS (during the pandemic) Subcategory: lack of staff
6.69 ± 1.71 out of 10, scoring significantly below the emergency department (5.57 ± 1.67)	Safety climate rating in service/unit	Workload	Nurse/patient ratio that, unlike other services, in the emergency department is not stable	Category: factors not favouring PS Subcategory: work overload
		Interruptions/distractions	Interruptions and distractions, in the emergency area related to the presence of family members	Category: factors not favouring PS Subcategory: interruptions and distractions
		Infrastructure	Having limited space but not restricting the number of patients accessing the service	Category: factors not favouring PS Subcategory: inadequate structural resources
		Workload	Caring for a single patient and performing one task at a time	Category: impact on PS during the pandemic
		Patient involvement	Individualised, person-centred care that supports patient engagement in patient safety	Category: factors favouring PS Subcategory: therapeutic relationship
		Technological resources	Considering technology as favouring PS and, in turn, as a risk factor	Category: factors considered ambivalently Subcategory: use of technology
		Material resources	The lack of material, technical and structural resources put PS at risk	Category: lack of material, technical and structural resources
82% of nurses had not reported any incident in writing during the last year, with significant differences regarding unit/service, work position, seniority and type of working day	Events reported	Training	Not knowing the existence of the notification system	Category: AE reporting system Subcategory: ignorance of its existence
		Notification system	Complex and laborious reporting system	Category: AE reporting system Subcategory: lack of time to complete it
		Confidence to report AE	The fact that the error to be reported was made by another team member slows down the notification	Category: motivation of the professional to report Subcategory: another pro’s Error
80% of nurse managers reported an incident compared to 15.4% of care nurses	Significant differences in bivariate analysis: work position and events Reported	Training	Knowing if what happened is an AE related to PS	Category: AE features Subcategory: types of AE that are not reported
		AE Types	Lack of awareness about what is considered an incident to report	Category: AE features Subcategory: types of AE that are not reported
		Leadership	Whether or not the notification system is anonymous	Category: AE reporting system Subcategory: anonymous Form

Source: prepared by the authors.
